# Effectiveness of photodynamic therapy on the treatment of chronic periodontitis: a systematic review during 2008–2023

**DOI:** 10.3389/fchem.2024.1384344

**Published:** 2024-05-16

**Authors:** Marzie Mahdizade Ari, Nour Amirmozafari, Atieh Darbandi, Roghayeh Afifirad, Parisa Asadollahi, Gholamreza Irajian

**Affiliations:** ^1^ Department of Microbiology, School of Medicine, Iran University of Medical Sciences, Tehran, Iran; ^2^ Microbial Biotechnology Research Center, University of Medical Sciences, Tehran, Iran; ^3^ Department of Microbiology, School of Medicine, Tehran University of Medical Sciences, Tehran, Iran; ^4^ Department of Microbiology, School of Medicine, Ilam University of Medical Sciences, Ilam, Iran

**Keywords:** PDT, photochemotherapy, dental scaling, chronic periodontitis, randomized controlled trials, systematic reviews

## Abstract

**Objective:**

This study investigated the effect of photodynamic therapy on chronic periodontitis patients and then evaluated the microbial, immunological, periodontal, and clinical outcomes. The significant effects of photodynamic therapy obtained by *in vitro* and *in vivo* studies have made it a popular treatment for periodontal diseases in recent years. Photodynamic therapy is a novel bactericidal strategy that is stronger, faster, and less expensive than scaling and root planing.

**Method:**

This study registered on PROSPERO (CRD42021267008) and retrieved fifty-three randomized controlled trials by searching nine databases (Medline, Embase, Scopus, Open Gray, Google Scholar, ProQuest, the Cochrane Library, Web of Science, and ClinicalTrials.gov) from 2008 to 2023. Of 721 records identified through database searches following title and full-text analysis, and excluding duplicate and irrelevant publications, 53 articles were included in this systematic review. Fifty of the 53 eligible studies fulfilled all the criteria in the Joanna Briggs Institute’s (JBI’s) Checklist for RCTs; the remaining articles met 9–12 criteria and were considered high quality.

**Results:**

The present study showed that photodynamic therapy in adjunct to scaling and root planing has the potential to improve periodontal parameters such as clinical attachment loss or gain, decrease in bleeding on probing, and probing pocket depth. In addition, photodynamic therapy decreases the rate of periodontal pathogens and inflammation markers, which, in turn, reduces the progression of periodontitis.

**Conclusion:**

Photodynamic therapy is considered a promising, adjunctive, and low-cost therapeutic method that is effective in tissue repair, reducing chronic periodontitis, reducing inflammation, and well-tolerated by patients.

## 1 Introduction

An inflammatory condition that affects the periodontium, cementum, and alveolar bone of the tooth is known as periodontitis. The global prevalence of periodontitis is estimated at 20%–50% (Garg et al., 2015). The primary cause of this disease is microorganisms that can be exacerbated by smoking and underlying disorders (diabetes and obesity) (Russo et al., 2016). In periodontitis, the attachments between the tooth-supporting tissue (periodontal tissue) and the tooth are destroyed, and this provides the basis for the emergence of periodontal pockets in which a wide range of periodontal pathogens can survive (Nazir et al., 2020). Chronic periodontitis (CP) refers to a long-term inflammatory status that is induced by a range of periodontal pathogens, such as *Porphyromonas gingivalis (P. gingivalis)*, *A. actinomycetemcomitans (Aggregatibacter actinomycetemcomitans),* and *Fusobacterium nucleatum (f. nucleatum),* etc., due to their virulence factors including enzymes, lipopolysaccharide (LPS), toxins, and heat shock proteins (HSPs), as well as biofilm formation. These virulence factors enable pathogens to destroy periodontal tissue and alveolar bone (Bassir et al., 2013; Meimandi et al., 2017). The highest incidence of CP is in older patients (82%), followed by adults (73%) and adolescents (59%) (Tadjoedin et al., 2017), and it seems that the severity of the disease increases with age (Meimandi et al., 2017). Periodontal pathogens can indirectly induce systemic disorders, such as diabetes and cardiovascular disease, by spreading directly from the damaged tissue and dental plaque in the oral cavity through the blood stream to other tissues and organs. Hence, the early treatment of this disease is very important (Nazir, 2017). Periodontal therapy involves scaling and root planing (mechanical debridement) and administration of antimicrobial agents, including antibiotics and mouthwashes (chemical therapy). Dental scaling refers to removing dental plaques (teeth tartar or dental calculus), which is sometimes combined with root planing, a process of smoothing root surfaces (Garg et al., 2015). Antibiotic resistance and lack of accessibility into the deeper areas of the periodontal pocket complicate treatment (Mahdizade-Ari et al., 2019; Amaral et al., 2020). While scaling and root planing (SRP) is still the gold standard of periodontal therapy (Jia et al., 2020), several new strategies have been developed to inhibit biofilm formation on the tooth surface.

One of the new therapeutic strategies is photodynamic therapy (PDT) (Meimandi et al., 2017), which is a non-invasive chemical method that first originated in 1990 for cancer therapy. PDT has the potential to treat various types of diseases, including microbial infections, by destroying abnormal cells (Garg et al., 2015). A light source and a photosensitizer (PS) are the two main components of PDT. Following the insertion of the photosensitizer at the site of infection, light is emitted at a specific wavelength, leading to the excitation of the photosensitizer from the ground state to the triplet state. The excited photosensitizer will produce two types of toxic reactive oxygen metabolites after an interaction with organic molecules: Type-Ⅰ (hydrogen peroxide, superoxide, and free hydroxyl radicals) and Type-Ⅱ (singlet-oxygen). This can lead to selective abnormal cell or microbial death (Kwiatkowski et al., 2018). The significant effects of photodynamic therapy on cellular and microbial populations have made it a popular treatment for periodontal diseases in recent years (Bassir et al., 2012; Campos et al., 2013; Kashef et al., 2016; Raut et al., 2018; Pooja and Mannava, 2020b; Joshi et al., 2020). The reduction of bacteria following the application of different photosensitizers in the treatment of chronic periodontitis was shown by both *in vitro* and *in vivo* studies. In comparison to SRP, photodynamic therapy is faster, less expensive, and has a stronger bactericidal effect (Jia et al., 2020). Moreover, due to the very low half-life of the singlet-oxygen, the antibacterial effect of PDT remains localized and limited to the treated sites (Joshi et al., 2020).

The latest update published by the American Academy of Periodontology and the European Federation of Periodontology recognized four stages (Ⅰ–Ⅳ) of periodontitis classified according to tooth loss and severity and include slight, moderate, chronic, and advanced (necrotizing) periodontitis (Kapoor et al., 2022). Slight and moderate chronic periodontitis is still affected by mechanical therapy through the removal of microbial plaques, but the chronic and advanced stage needs new treatment to penetrate deep into the periodontal pockets (Swedish Council on Health Technology Assessment SBU, 2004). Chronic periodontitis was chosen in the present study because it is more common among the population, and it is important to diagnose and treat it in order to suppress its progression to advanced periodontitis. Therefore, the aim of this systematic review was to investigate more than 10 years of clinical trials (2008–2023) to determine the effectiveness of PDT in adjunct to the scaling method as a simple method for the treatment of chronic periodontitis.

## 2 Materials and methods

### 2.1 Guidelines

The Preferred Reporting Items for Systematic Reviews and Meta-Analyses (PRISMA) guidelines (2020) were used to conduct this systematic study (Page et al., 2021). This systematic review has been registered in PROSPERO (international prospective register of systematic reviews): CRD42021267008 (www.crd.york.ac.uk/PROSPERO/display_record.php?Recordid=267008).

## 3 Population (P), intervention (I), comparison (C), and outcomes (O)—PICO

1. **Population:** Patients diagnosed with chronic periodontitis
2. **Intervention:** PDT—monotherapy or as an adjunct to SRP
3. **Comparison:** SRP alone or SRP + Antibiotic therapy
4. **Outcome:** Periodontal parameters and/or microbiological and/or immunological profiles
5. **Study design:** Randomized controlled trials

### 3.1 Main research question

“Is photodynamic therapy in combination with SRP effective in improving periodontal parameters and microbiological and immunological profiles in patients with chronic periodontitis?”

### 3.2 Search strategy and information sources

**FIGURE 1:**
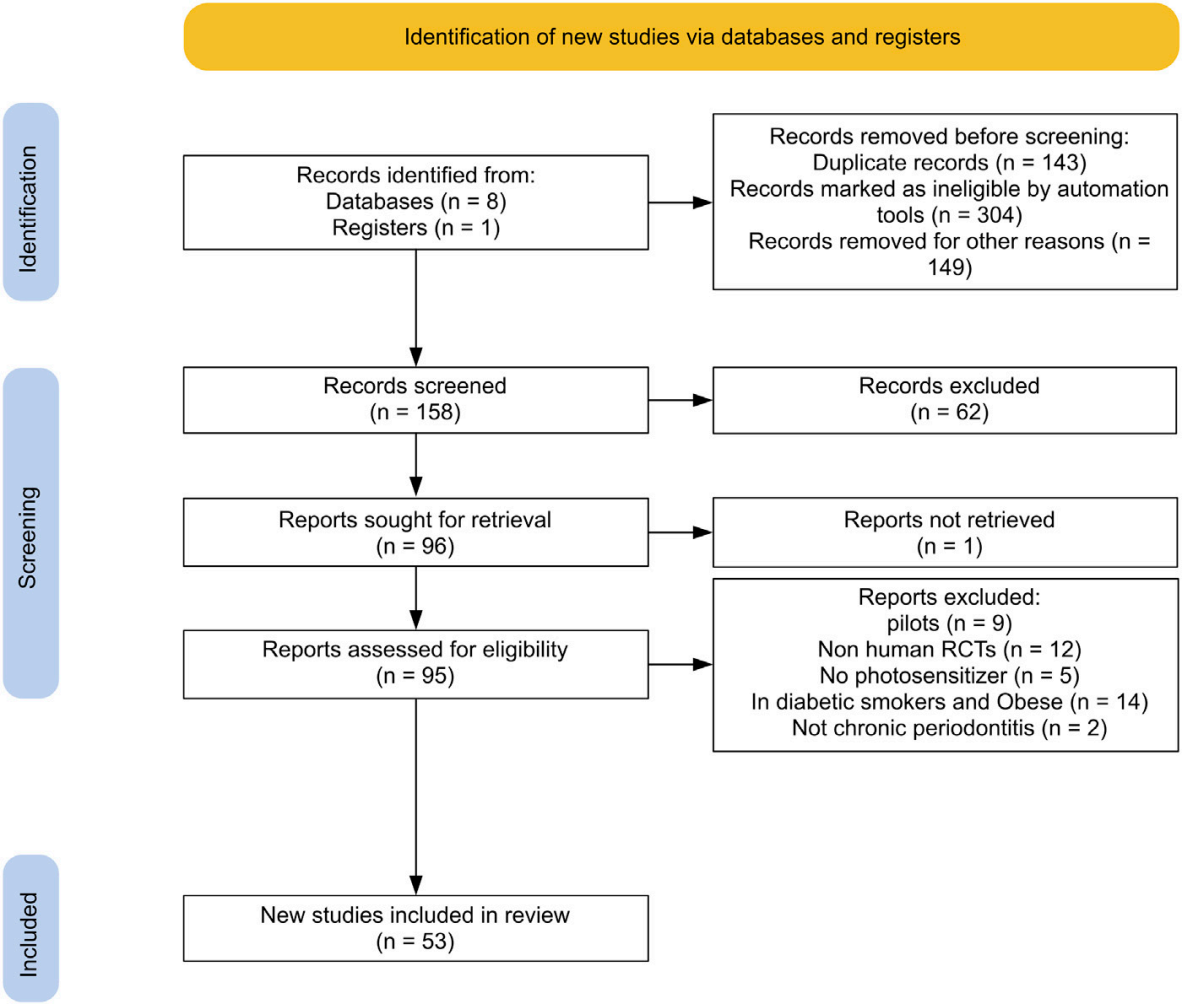
PRISMA flowchart of the search strategy process.

In this review, international databases (Medline, Embase, Scopus, Open Gray, Google Scholar, ProQuest, the Cochrane library, and Web of Science) were searched for eligible articles published in English from February 2008 to January 2023. In addition, clinical trial registries for ongoing or recently completed trials (clinicaltrials.gov) were searched. The search strategy was performed by a combination of the following terms: “photodynamic therapy” OR “photochemotherapy” AND “chronic periodontitis” AND “dental scaling” AND “randomized clinical trial”. Two separate reviewers screened the titles and abstracts of each study. Finally, the full text of articles deemed to be potentially eligible was retrieved for further detailed evaluation. To identify additional relevant publications, we also searched the reference lists of involved articles and relevant reviews for inclusion in this study. Disagreements were resolved by a third reviewer. Duplicates were removed by End Note 20. Figure 1 illustrates the flow diagram of the search and article selection process.

## 4 Eligibility criteria

### 4.1 Inclusion criteria

The following items were considered inclusion criteria in this study.

1. Randomized controlled trials
2. Trials with well-described and defined outcomes
3. Trials carried out between 2008 and 2023

### 4.2 Exclusion criteria

The following exclusion criteria were applied in this study.

1. The RCT incorporated volunteers with systemic disease (e.g., diabetes, obesity) or any situation that could affect PDT outcomes (smokers, etc.).
2. Trial using laser only, without any photosensitizer
3. Trials on non-human cases
4. Trials written in a language other than English
5. Trials that did not provide enough information about the outcomes
6. Review, systematic review, and case reports
7. Duplicate trials

### 4.3 Systematic review outcomes and data analysis

Primary outcomes of the present systematic review were the effects of PDT on bleeding on probing (BOP), clinical attachment level (CAL), probing pocket depth (PPD), gingival index (GI), and plaque index (PI), as well as microbiological and immunological parameters. Secondary outcomes were types of photosensitizers and the concentrations and characteristics of lasers used in PDT protocol to control chronic periodontitis. Data were analyzed by considering *p* < 0.05 as statistically significant.

### 4.4 Risk-of-bias assessment

The quality assessment of the RCTs was evaluated independently by two authors prior to inclusion in the review using the Joanna Briggs Institute tool (JBI, 2014) (Aromataris, 2020). A score ranging from 0 to 13 points was attributed to each study. Items in the risk-of-bias (RoB) tool were scored with “yes” (low risk of bias), “no” (high risk of bias), or “unclear or unapplicable” (indicating that the item was not reported, and therefore, the risk of bias was unknown). Reporting quality was evaluated by screening all manuscript sections. Supporting information is presented in Table 3.

## 5 Results

### 5.1 Search results

Of 721 records identified through database searches, 53 articles were retained to be included in this systematic review following title and full-text evaluation and excluding duplicate and irrelevant publications (Figure 1).

### 5.2 Characteristics of the studies

The average age of the volunteers in the trials who underwent PDT, with or without SRP treatment, was 47.93 years old. According to Table 1, most PDT studies were conducted in Brazil (17 of the 53 studies and 328 of the 1878 patients) and India (13 of the 53 studies and 409 of the 1878 patients), respectively. Moreover, as shown in Table 2, PDT treatment was most commonly used during 2019 in different clinical trials. Data regarding microbiological analysis and periodontal and immunological parameters are shown in Table 3. A number of ongoing trials obtained from the ClinicalTrials.gov database have not yet reported their results, possibly because the studies are incomplete or the executors have not updated their findings in the ClinicalTrials.gov database.

**TABLE 1:** Countries that commonly used PDT during 2008–2023; other countries include France, Thailand, Jordan, Italy, Austria, Malaysia, Pakistan, Spain, Slovenia, China, Greece, Netherlands, Turkey, and Singapore.

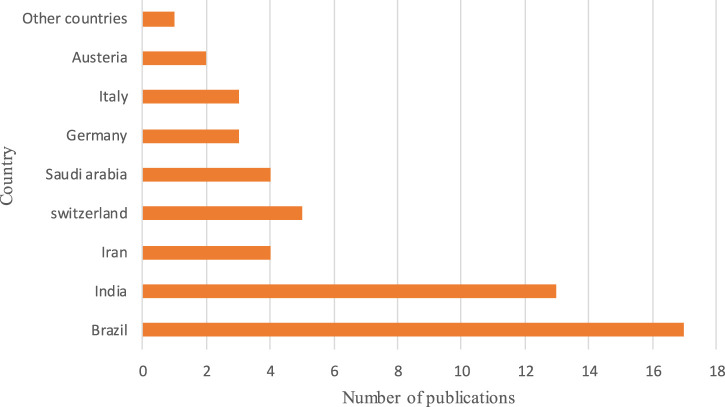

**TABLE 2:** Years of common application of PDT during 2008–2023.

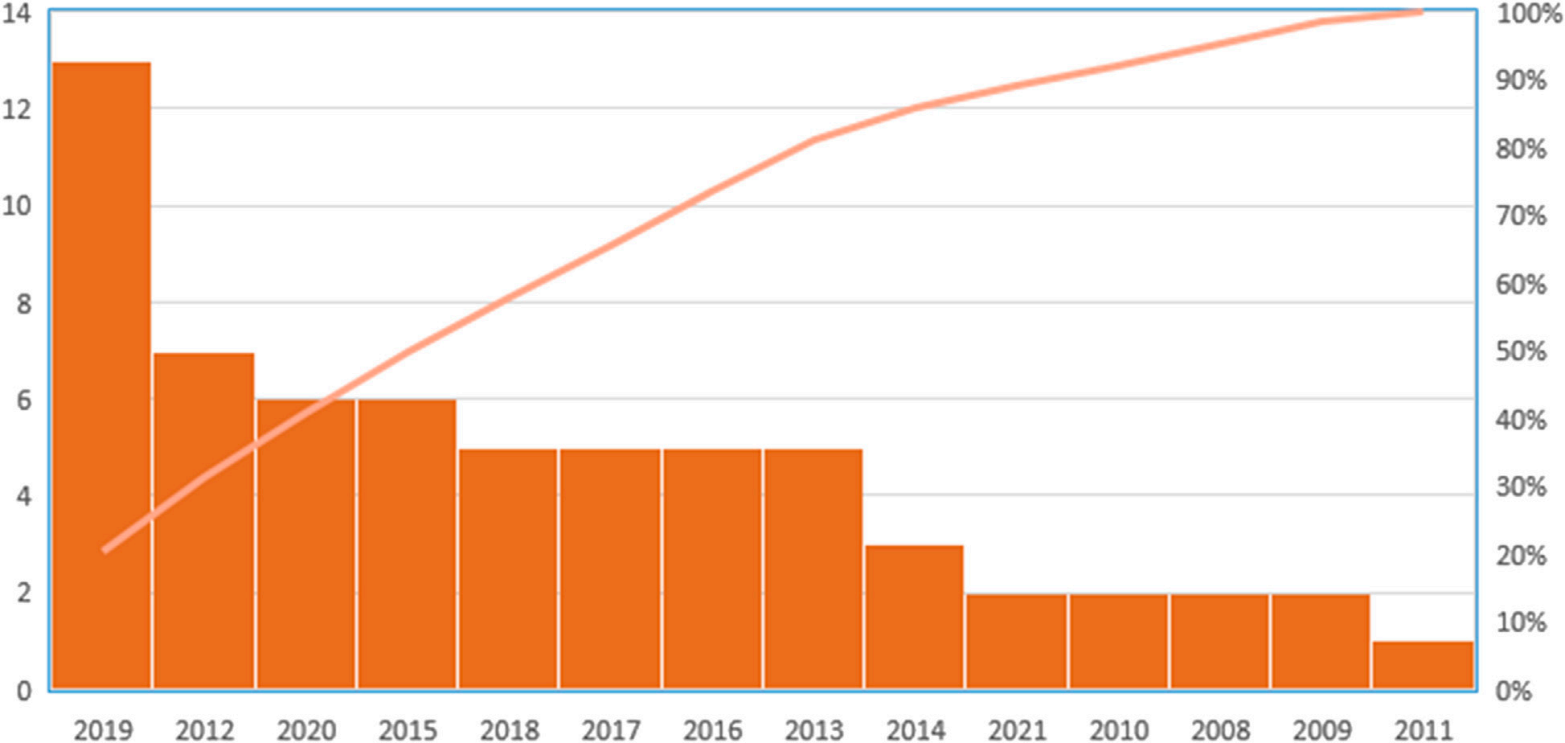

**TABLE 3:** Design and demographic characteristics of the included studies. Abbreviations: T-C, treatment–control groups; F/M, female–male; w/v %, weight/volume; WL, wave length; sec, second; min, minutes; MO, month; W, week; PO, power output; PD, power density; LD, laser diode; GaAlAs, gallium–aluminum–arsenide; InGaAlP, indium gallium–aluminum–phosphide; LLLT, low-level laser therapy; LT, laser therapy; SRP, scaling and root planing; MB, methylene blue; PTZ, phenothiazine chloride; ICG, indocyanine green; TB, toluidine blue; AlClFc, chloro-aluminum phthalocyanine; SP, *Salvadora persica*; CP, chronic periodontitis; GCP, generalized chronic periodontitis; CPUT, chronic periodontitis untreated; T2D, Type 2 diabetes; PI, plaque index; BOP, bleeding on probing, AL, clinical attachment loss; PPD, probing pocket depth; NSPT, non-surgical periodontal therapy; HbA1C, glycated hemoglobin; GBI, gingival bleeding index; RAL, relative attachment level; PSs, probing depths; RC, gingival recession; FMPS, full-mouth plaque score; FMBS, full-mouth bleeding score; TBC, total bacteria counts; SFFR, sulcus fluid flow rate; mSBI, modified sulcular bleeding index; PGM, position of the gingival margin; RCAL, relative clinical attachment level; IL, interleukin; MMP, matrix metalloproteinase; PS%, plaque scores; PBI, papilla bleeding index; FMD, full-mouth disinfection; LDH, lactate dehydrogenase; TNF, tumor necrosis factor alpha; RANK-L, receptor activator of nuclear factor-kappa B ligand; OPG, osteoprotegerin; PMN, polymorphonuclear leukocytes; RBC, erythrocytes; DEC, damaged epithelial cells; Pi, *Prevotella intermedia*; Pg, *Porphyromonas gingivalis*; Td, *Treponema denticola*; F.n, *Fusobacterium nucleatum*; ERL, erlotinib; LED, light-emitting diode; MBS, methylene blue-sodium dodecyl sulfate solution; ST, surgical periodontal treatment; OFD, open-flap debridement; REC, recession; KTP, potassium titanyl phosphate; OFD, open-flap debridement; AV, aloe vera.

First author, year, and country (clinical gov identifier)	Sample size (f/m) and mean age	Study design	Participant characteristics	Photodynamic therapy			Parameters measured	Treatment arms and follow-up	Conclusions
				Photosensitizer and con		Light source and irradiation time			
Haider A. Alwaeli (2015) Jordan	16 (103/33) 39.4 y	RCT	CPUT	PTZ	10 mg/ml	LD 10 s WL: 670 nm	PPD, CAL, and BOP	SRP/PDT Baseline, after 3, 6, and 12 months	• Significant reduction and increase in PPD, BOP, and CAL, respectively
									• No adverse effects from PDT
Seyed H. Bassir1 (2013) (NCT01330082) Iran	16 (8/8) 50.3 ± 8.7 y	RCT DB	CP (moderate, severe)	TBO	0.1 mg/ml	LED 10 s WL: 625–635 nm	BOP, PPD, and CAL	LED/PDT/SRP Baseline, after 1 and 3 months	• Significant improvements in all clinical parameters
Betsy Joseph (2014) (REFCTRI2010006105) India	88 (51/39) 39.6 ± 8.7 y	RCT DB	CPUT	MB	10 mg/ml	LD 60 s WL: 655 nm	PPD, CAL, GI, GBI, and halitosis	SRP/PDT Baseline, after 2 weeks, and 3 months	• Significant reduction in PPD and CAL
									• Significant improvement in GI and GBI after 2 weeks and 1 month
									• Less improvement in GI and GBI after 3 months and in PI at 2 weeks
									• Significant difference in halitosis after 1 month (not long-lasting)
Braun A (2008) Germany	20 (11/9) 46.6 ± 6.1 y	RCT	CPUT	PTZ	10 mg/ml	LD 0.25 s WL: 660 nm	SFFR, BOP, RAL, PD, and GR	SRP/PDT Baseline, after 1 week and 3 months	• Significant reduction in RAL, PD, SFFR, and BOP after 3 months
									• GR increased 3 months after treatment with and without adjunctive PDT
Pitchanun Bundidpun (2017) Thailand	20 (13/7) 47.25 ± 8.91 y	RCT	CP (moderate- severe)	PTZ	10 mg/ml	LD 10 s WL: 660 nm	PPD, CAL, PI, GBI, and GI	PDT/control Baseline, 1 month, 3 months, and 6 months	• Significant improvement in all periodontal parameters in both groups
									• Significant differences of GBI and GI at 3 and 6 months in the test group
									• No significant differences of PD, CAL, and PI in the test group
Chetan Purushottam Raut (2018) India	50 (22/28) 49.8 ± 5.10 y	RCT	CP	ICG	5 mg/mL	LD 60 s WL: 810 nm	PI, BOP, PPD, CAL, and microbiological analysis	SRP/PDT Baseline and 6 months	• Significant reduction in PD, CAL, and BOP
									• Non-significant change in the intergroup comparison of PI
									• Significant reduction in microbiological analysis
Marco Giannelli (2012) Italy	26 (11/15) 46.7 y	RCT DB	CP	MB	0.3% w/v in water	(PAPD): GaAlAs, and InGaAlP 60 s WL: 810 nm and 635 nm	PPD, CAL, BOP, PMN, RBC, DEC, and microbiological analysis	SRP/PDT 0 day-, 15 days, 30 days, 45 days, 60 days, 75 days, 90 days, and 365 days	• Significant reduction in PD, CAL, BOP, bacterial contamination, especially spirochetes, PMN, and RBC shedding in the gingival samples
Marco Giannelli (2015) Italy	24 (11/13) 46.1 y	RCT DB	CP	MB	0.3% w/v	(PAPD): GaAlAs (PAPD), and InGaAlP 60 s WL: 810 nm and 635 nm	PPD, CAL, BOP, PMN, RBC, DEC, and microbiological analysis	PAPD + SRP/SRP 4 years follow-up	• Significant improvement in PD, CAL, and BOP, and bacterial contamination and PMN-RBC shedding in the exfoliative sample in PAPD + SRP
Gisele N. Campos (2013) Brazil	13 (6/7) 48.15 ± 7.53 y	RCT DB	CP	MB	10 mg/ml	LD 60 s WL: 660 nm	PPD, CAL and BOP, PGM, FMPS, and FMBS	SRP/PDT Baseline and 3 months	• Significant reduction in the number of sites with PPD <5 mm and also CAL without BOP after 3 months
K. Grzech-Leśniak (2019) Switzerland	40 (25/15) 50.3 ± 11.6 y	RCT DB	GCP	TB	0.1% w/v	LD 30 s WL: 635 nm	FMPS, BOP, PPD, CAL, RC, and microbiological analysis	SRP/PDT Baseline, 3 months, and 6 months	• Significant improvement in FMPS, PPD and CAL values but no significant changes for RC
									• Significant reduction in TBC and BOP
									• Significant decrease in the number of all bacteria except A.a, Pg, Td, and Tf
Kinga Grzech-Leśniak (2018) Switzerland	84 (38/46) 48.6 ± 9.4 y	RCT DB	GCP	TB	0.1% w/v	LD 30 s WL: 635 nm ERL Energy: 40 mJ frequency: 40 Hz	PD, RC, PI, BOP, AT, tissue injury test, and microbiological analyses	SRP/SRP + PDT/ERL 1 week, 3 months, and 6 months	• PI and BOP decreased in ERL
									• PPD decreased in all groups
									• Reduction in some bacteria after follow-up
									• No signs of carbonization or teeth injury
Greta Hill (2019) Germany	20 (17/3) 61.1 y	RCT DB	CP	ICG	0.1 mg/ml	LD 60 s WL: 808 nm	BOP, SFFR, RAL, PD, GR, and microbiological analysis	SRP/PDT Baseline, 2 weeks, 3 months, and 6 months	• Significant reduction in BOP, RAL, and PD in both groups
									• Significant reduction in SFFR in the test group
Israel Alexandre de Arau´ jo Sena (2019) Brazil	9 (NA) >18y	RCT	CP	AlClFc	5 µM	LD 15 s WL: 660 nm	PI, BOP, PPD, and CAL	SRP/PDT Baseline and 3 months	• Decrease of BOP and PD and clinical insertion gain in both groups
Karuna Joshi (2019) (CTRI/2017/11/010638) India	29 (15/14) 30 - 60 y	RCT	GCP (moderate, severe)	ICG	1 mg/ml	LD 30 s WL: 810 nm	PI, mSBI, PPD, and CAL	SRP/PDT Baseline and 3 months	• Significant reduction in PI and mSBI in both groups
									• Significant improvement in PPD and CAL in the ICG-group
Maria F. Kolbe (2014) (NCT01670305) Brazil	22 (12/10) 48.52 y	RCT	CP	MB	10 mg/mL	LD 60 s WL: 660 nm	PGM, RCAL, PPD, BOP, FMPS, and FMBS, and microbiologic and cytokine profiles	SRP/PDT Baseline, 3 months, and 6 months	• Improvements in clinical parameters, except BOP was not reduced
									• Lower levels of A.a and Pg in both groups
									• Increased levels of IL-4 and reduced IL-1b and IL-6
Kaveri Kranti Gandhi (2019) India	30 (NA) 30–60 y	RCT	(Moderate-severe) CP	ICG	NA	LLLT and PDT LD 60 s WL: 810 nm	GI, PPD, CAL, and microbiological analysis	SRP/SRP+PDT/SRP+LLLT Baseline, 1 month, 3 months, 6 months, and 9 months	• Significant reduction in GI, PPD, CAL, Pg, and A.a in test groups
Suryakanth Malgikar (2017) India	24 (9/15) 24–55 y	RCT	CPUT	MB	1% w/v	LD 10–30 s WL: 980 nm	PI, GI mSBI, PPD, and CAL	SRP/SRP+PDT/SRP+PDT+LLLT Baseline, 1 month, 3 months, and 6 months	• Significant improvements for all variables
Fayez Hussain Niazi (2020) Saudi Arabia	73 (NA) 47.56 y	RCT	CP	ICG	NA	LD 60 s WL: 810 nm	PI, BOP, PPD CAL, IL-6, and TNF-α	SRP+PDT/SRP+SP/SRP Baseline, 3 months, and 6 months	• Significant improvement in the BOP in Group II
									• CAL increase in Group-I
									• Reduced levels of IL-6 in Group- I and II
									• TNF-α significant decrease in Group II
Raoul Polansky (2009) Austria	58 (36/22) 48.7 y	RCT	CP	HELBO Blue	NA	LD 60 s WL: 680 nm	GI, BOP, CAL, and microbiological analysis	PDT/control Baseline, 10 days, 42 days, and 90 days	• Significant reduction of Pg
									• Significant reduction in clinical parameters in all groups
Reza Pourabbas (2014) (IRCT2012121611770N1) Iran	24 (14/10) 46 ± 8 y	RCT	CP (moderate-severe)	TB	NA	LD 120 s WL: 638 nm	PPD, BOP, CAL, RC, IL-6, TNF-α and MMP8-9, and PMNs	SRP/SRP+PDT Baseline and 3 months	• Significant improvements for all variables
									• Reduction of PMNs for all patients
SJ Pulikkotil (2016) Malaysia	20 (7/13) 45.2 ± 6.7 y	RCT	CP	MB	NA	LED 10 s WL: 650 nm	PPD, CAL, BOP, PS%, and microbiological analysis	SRP/SRP+PDT Baseline,1 month, and 3 months	• Significant clinical improvement
									• BOP reduced significantly only in the test group
									• No difference in the count of Aa was detected between the groups
Reza Birang (2015) (IRCT2015021021029N1) Iran	20 (13/7) 37.2 ± 8.6 y	RCT	CP	Emundo	NA	LT, and PDT LD 10 s, 15 s, and 25 s WL: 810 nm	PPD, CAL, PBI, and microbiological analysis	SRP/SRP+LT/SRP+PDT Baseline, 6 weeks, and 3 months	• Significant improvements for CAL gain, PPD reduction, PBI, and microbial count
Kura Srikanth (2015) (NCT02043340) India	30 (NA) 30-55 y	RCT	CP	ICG	5 mg/ml	LD 5 s WL: 810 nm	LDH, PI, RC, PPD CAL, and microbiological analysis	SRP/SRP+ LD SRP+PDT Baseline, 1 week, 12 weeks, and 24 weeks	• Significant decrease in bacteria in PDT
									• ICG application does not cause to tissue damage and differences in LDH levels
Leticia Helena Theodoro (2012) Brazil	33 (21/12) 43.12 ± 8.2 y	RCT	CP	TBO	100 μg/ml	LD 150 s WL: 660 nm	PI, BGI, BOP, PPD, RC, CAL, and microbiological analysis	SRP/SRP+TBO/SRP+PDT Baseline, 60 days, 90 days, and 180 days	• Improvement in all clinical parameters in all treatment groups but no significant difference in periodontal parameters among the groups
									• Significant reduction of periodontopathogens by PDT treatment
Letícia Helena Theodoro (2017) Brazil	34 (16/18) 47.55 ± 7.55 y	DB RCT	CP	MB	10 mg/ml	LD 48 s WL: 660 nm	BOP, PD, and CAL	MTZ+AM X +SRP/PDT+SRP Baseline and 90 days	• Significant improvement in CAL in the PDT group
									• Reduction of both BOP and the residual pockets in both groups
Segarra -Vidal M (2017) (NCT01532674) Spain	37 (26/11) 55 ± 2 y	RCT	GCP (moderate-advanced)	MB	0.005% w/v	LD 60 s WL: 670 nm	PI, PPD, RC, CAL, BOP, GSF volume, and microbiological analysis, as well as IL-1β, IL-6, TNF-α, RANK-L, and OPG	SRP/SRP+PDT/healthy Baseline, 5 weeks, 13 weeks, and 27 weeks	• Significant improvements for clinical parameter
									• Significant decrease in RANK-L and A.a count in the SRP+PDT group
Walia Pooja (2019) India	40 (34/6) > 18 y	RCT	Moderate CP	MB	1% w/v	LD ≤30 s WL: 940 nm	GI, PPD, CAL, and microbiological analysis	SRP/SRP+PDT Baseline, 1 month, and 3 months	• Significant GI scores in the test group
									• Non-significant reduction in PPD and CAL in SRP+PDT
									• Non-significant reduction in counts of Aa, Pg, and Pi in the test group
Goh Xian Jun Edwin (2013-2016) (NCT02666573) Singapore	27 (16/11) 55.5 ± 7.9 44-70 y	RCT	CP	TBO	0.1 mg/ml	LED s WL: 630 nm	PPD, CAL, BOP as well as IL-1β, IL-6 -8, TNF-α, and MMP-8	SRP/SRP+PDT Baseline, 3 months, and 6 months	• Significant reduction in CAL and PPD at 3 months
									• No significant differences at 6 months
Alparslan Dilsiz (2013) Turkey	24 (14/10) 30-58 40.7 ± 7.3 y	RCT	CP	MB	1%	LD, and KTP laser LD: 60 s WL: 808 nm	PI, GI, BOP, PD, and CAL	PDT/KTP/SRP Baseline and 6 months	• Significant improvements in BOP and PD decrease and CAL gain
						KTP: 30 s WL: 532 nm			
Letícia H. Alvarenga (2019) Brazil	20 (NA)	RCT	CP	MB	1000 μM	LD: 4, 3, and 5 min WL: 660 nm	Microbiological analysis	PDT-MB/PDT-MBS	• Significant microbial reduction levels with 5 min of irradiation
Bernd W. Sigusch (2010) Germany	24 (17/7) 42.66	RCT	CP	PTZ	NA	LD 60 s WL: 660 nm	PI, PD, BOP, GR, CAL, reddening, and F.n concentration	PDT/SRP Baseline, 1 week, 4 weeks, and 12 weeks	• Significant reduction of F.n, reddening, PD, BOP, and CAL
Uislen B. Cadore (2018) Brazil	16 (NA)	RCT	CP	PTZ	10 mg/mL	LD 60 s WL: 660 nm	CAL, PD, GR, BOP, PI, and microbiological analysis	PDT/ST 0, 2 days, 7 days, and 14 days	• Significant reduction in PD
									• Changes in the subgingival microbiota were similar between the groups
Nicos Christodoulides (2008) Netherlands	24 (7/17) 45 ± 8.11 y	RCT	CP	HELBO Blue	NA	LD 60 s WL: 670 nm	FMPS, FMBS, PD, GR, CAL, and microbiological analysis	PDT/SRP Baseline, 3 months, and 6 months	• Significant greater improvement in FMBS
Monica Grazieli Correa (2016) Brazil	15 (NA)	RCT	P	MB	10 mg/ml	LD 60 s WL: 660 nm PD: 60mW	PPD, BOP, and microbiological analysis	PDT/SRP Baseline and 3 days, 7 days, 14 days, and 90 days	• Reduction of Aa levels for a short-term period
Snehal A. Dalvi (2019) India	20 (14/6) 30-55 y	RCT	CP	ICG	1 mg/ml	LD 30 s WL: 810 nm	PPD, RAL, RGML, PI, GI, and GBI	PDT/OFD Baseline and 3 months	• Significant improvement in RAL, RGML, and GI
Catherine Giannopoulou (2012) Switzerland	32 (23/9) 52 y	RCT	CP	PTZ	100 mg/mL	PDT: LD 60 s WL: 660 nm	PD, BOP, REC, and cytokines and acute-phase proteins	PDT/DSL (or LD) and SRP	• Significant change in the level of cytokines and acute-phase proteins
						LD: 60 s WL: 810 nm			
Lui J (2011) China	24 (14/10) 50 y	RCT	CP	MB	1%	LD: 5–10 s WL: 940 nm	Plaque, BOP, PD, GR, and interleukin-1b levels	PDT/SRP Baseline, 1 month, and 3 months	• Significant decrease in interleukin-1b levels, BOP, and PD
Shaswata Karmakar (2020) India	20 (NA) 35–55 y	RCT	CP	ICG	1 mg/ml	LD 30 s WL: 810 nm	PD, CAL, and microbiological analysis	PDT/SRP Baseline and 3 months	• Significant improvement in PD and CAL levels
									• Non-significant differences between microbiological parameters between groups
Fotios Katsikanis (2019) Greece	21 (13/8) 48.2 ± 8.2 y	RCT	CP	MB	1%	PDT (GaAlAs diode laser) 2 min WL: 670 nm	PD, CAL, BOP, and PI	PDT/LD/SRP Baseline, 3 months, and 6 months	• Significant improvements in PD and BOP
						LD: 30 s WL: 940 nm			
Luchesi VH (2013) Brazil	37 (70% female) 50.49 y	RCT	CP	MB	10 mg/ml	LD 60 s WL: 660 nm	CAL and microbiological and cytokine analysis	PDT/only PS groups Baseline, 3 months, and 6 months	• Decrease in P.g and TF, and GM-CSF, IL-8, IL-1β, and IL-6 levels only in the PDT group
									• Improvement in clinical parameters after both therapies
Marco Giannelli (2018) Italy	24 (NA)	RCT	CP	TBO	0.1%	LED 60 s WL: 635 nm	clinical and cytofluorescent periodontal markers	iPAPD/iPAPD+SRP	• Significant reduction in PD, BOP, bacteria, PMN, and damaged ep cells
Martina Lulic (2009) Australia	10 (3/7) 54 y	RCT	CP	PTZ	NA	LD 60 s WL: 670 nm	PI, PPD, BOP, and CAL	PDT/SRP 3 months, 6 months, and 12 months	• Greater PPD reductions, significant CAL gain, and significant decreased BOP
Mohammad Berakdar (2012) Iran	22 (10/12) 59.3 ± 11.7	RCT	CP	MB	0.005%	LD 60 s WL: 670 nm	BOP, PI, PD, and CAL	PDT/SRP Baseline (one week before therapy), 1 month, 3 months, and 6 months	• Greater reduction in the PD
Claudio Mongardini (2012) Italy	30 (7/13) 46.2 y	RCT	CP	TBO	0.1 mg/ml	LED WL: 628 nm	BOP, PPD, and microbiological analysis	PDT/SPT (SRP)	• Higher reductions of red complex bacteria
									• Decrease in PPD levels
Milan Petelin (2015) Slovenia	27 (NA)	RCT	CP	PTZ	NA	LD 60 s WL: 660 nm	BOP, PPD, CAL, and microbiological analysis	PDT/SRP Baseline, 1 week, 3 months, and 6 months	• Higher reduction of BOP
									• PDT reduce TF, TD, and A.a, significantly
Sartaz Rahman (2020) India	15 (7/8) 35–60 y	RCT	CP	ICG	NA	LD 60 s WL: 810 nm	API, PBI, PPD, RAL, and microbiological analysis	PDT/SRP Baseline and 3 months	• Significant reduction in clinical parameters and Pg
Russo C 2016 Rome	11 (7/4) 37–67 y	RCT	CP	Helbo Blue	NA	LD 10 s WL: 670 nm	PD	PDT/SRP	• Significant improvement in reduction of PD
Dorothee Sch¨ar (2020) Switzerland	39 (17/9) 59.0 ± 10.5 y	RCT	CP	PTZ	1.0 %	LD 60 s WL: 670 nm	BOP, PPD, and CAL	PDT/SPT (SRP) Baseline, 3 months, and 6 months	• Significant reduction in BOP, improvement of PPD, and CAL
Saurabh H Shingnapurkar (2016) India	(NA) 25–55 y	RCT	CP	ICG	1 mg/ml	LD 810 nm	PI, GI, PPD, and RAL	PDT/SRP Baseline, 1 month, and 3 months	• Improvement of PPD and RAL
Davi Neto de Ara´ ujo Silva (2020) Brazil	22 (NA) over 18 y	RCT	CP	AlClPc	0.5mL	15 s WL: 660 nm	GSH, MDA, PI, GBI, BOP, PD, and CAL	PDT/SRP Baseline, 3 months, and 6 months	• Decreased BOP, PD, CAL, and MDA
Kanchana Sukumar (2020) India	33 (8/22) 38.60 ± 6.75 y	RCT	CP	ICG	1mg/ml	LD 30 s WL: 810 nm	PPD, CAL, PI, GI, GBI, and microbiological analysis	PDT/SRP Baseline, 1 week, 2 weeks, and 4 weeks	• Significant improvement in all clinical parameters
									• Significant greater reduction in P.g, A.a, T.f, F.n, and Td
Ananya Wadhwa (2021) India	30 (8/22) 46 ± 7.1 y	RCT	GCP	TBO	250 μg/ml	LD 5 s WL: 810 nm	GI, PI, SBI, PPD, RAL, and microbiological analysis	PDT/SRP Baseline, 3 months, and 6 months	• Significant improvement in all clinical and microbiological parameters
Zeeshan Qamar (2021) Saudi Arabia	150 G1: (19/31) 46.76 ± 8.2 y G2: (22/28) 48.34 ± 6.7 y G3: (17/33) 51.02 ± 9.4 y	RCT	CP	ICG	NA	LD WL: 810 nm PD: 100 mW	PI, BOP, PPD, and CAL, and IL-6, IL-8, and TNF-α	SRP (G1)/SRP+PDT (G2)/SRP+AV (G3) Baseline, 3 months, and 6 months	• Significant improvement in all clinical parameters and cytokines

### 5.3 Study quality and risk of bias

**FIGURE 2:**
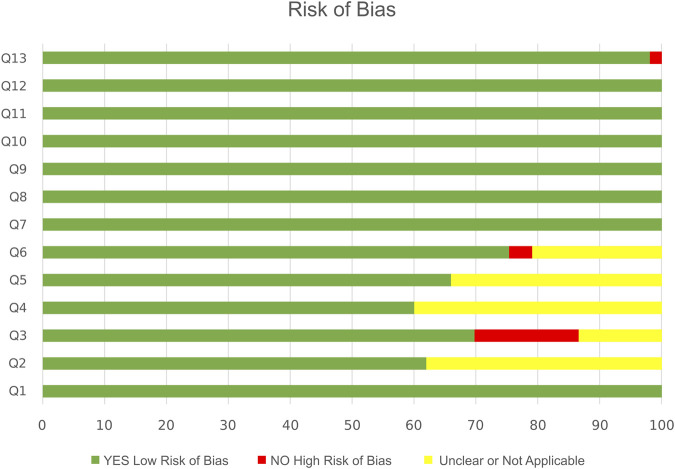
Risk of Bias of RCTs according to the JBI tool.

All the articles were RCTs, based on the definition used by the Joanna Briggs Institute’s (JBI’s) critical appraisal tool (Aromataris, 2020). Fifteen of 53 eligible studies fulfilled all the criteria in the JBI Checklist for Randomized clinical trials; the rest met nine to twelve criteria and were considered high quality. All studies were well designed and carried out. Twelve of 53 articles (22.6%) declared blinding of investigators (Q2) and outcome accessor (Q6). The JBI RoB results are elaborated in Figure 2.

### 5.4 Treatment procedure (protocol) of photodynamic therapy

**FIGURE 3:**
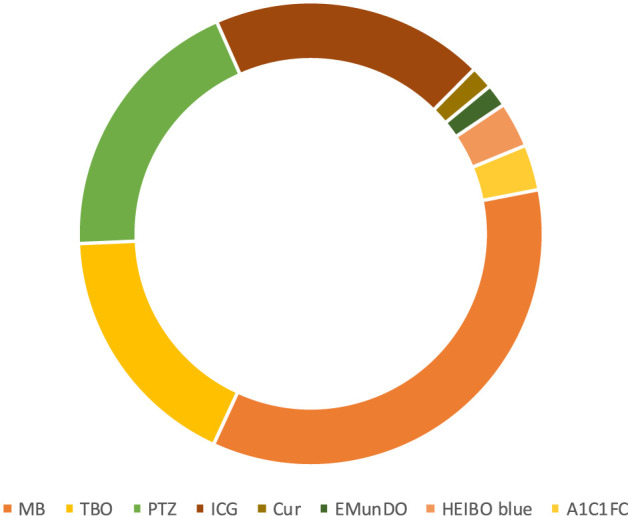
Most commonly used photosensitizers by trials during 2008–2023. Methylene blue, phenothiazine chloride, toluidine Blue O, and indocyanine green were the most frequently used photosensitizers.

**FIGURE 4:**
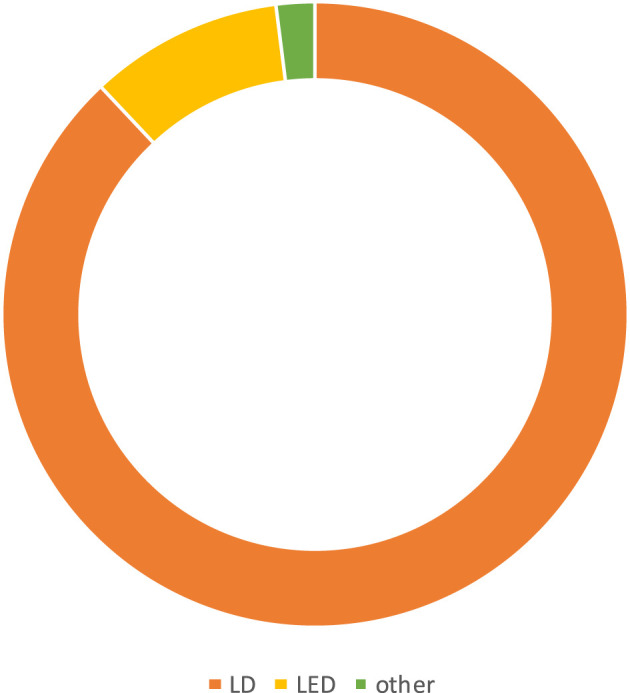
Most commonly used light sources by trials during 2008–2023. LD and light-emitting diode (LED) were the most prevalent light sources for the excitation of PS in PDT.

The most common photosensitizer used in the PDT procedure in different trials was methylene blue (MB) (41.50%) (Figure 3). Phenothiazine chloride (PTZ) (22.64%), toluidine blue O (TBO), and indocyanine green (ICG) (20.75%) were the next-most common photosensitizers. Uncommon photosensitizers, such as curcumin (Cur), EMUNDO, HELBO Blue, and A1C1FC, were also used. As shown in Figure 4, 45 of the 53 clinical trials (83.33%) used a laser diode (LD) to excite the PS in the photochemical process of PDT. Generally, wavelength ranges of 465–485 nm (Joshi et al., 2020) to 980 nm (Malgikar et al., 2016) were used in different trials, and 660 nm was the most frequently used laser diode wavelength. Because of heterogeneity in the characteristics of laser beams used in different trials, including heterogeneity in laser density energy, it was not possible to compare the findings of all the studies.

The following information is a summary of the methods used to deliver PS in the protocols included in the PDT. In all RCTs, the periodontal pocket area was rinsed with water, and then, the PS was placed topically in the bottom of the pocket by using a disposable Luer syringe or blunt needle so that the PS completely filled the bottom of the pocket without forming a bubble. Then, PS was irradiated in a specific range (Alwaeli et al., 2015) after 0.25, 10 s (Queiroz et al., 2013; Dos Santos et al., 2016; Pulikkotil et al., 2016; Grzech-Lesniak et al., 2019; Harmouche et al., 2019; Hill et al., 2019), and 30 s (8 out of the 53 studies), as well as 1 (Polansky et al., 2009; Bassir et al., 2012; Cappuyns et al., 2012; Theodoro et al., 2012; Alwaeli et al., 2013; Campos et al., 2013; Dilsiz et al., 2013; Betsy et al., 2014; Queiroz et al., 2015; Barbosa et al., 2016; Theodoro et al., 2017; Barbosa et al., 2018; AlAhmari et al., 2019; Gandhi et al., 2019; Ivanaga et al., 2019; Pooja and Mannava, 2020b; Joshi et al., 2020; Niazi et al., 2020), 2 [50] 3 min (Raut et al., 2018; Pooja and Mannava, 2020a), and 5 min (Sena et al., 2019).

Almost all (52) studies used PDT as an adjunct to SRP for the management of chronic periodontitis, and five studies used methods other than SRP along with PDT (Polansky et al., 2009; Giannopoulou et al., 2012; Dilsiz et al., 2013; Petelin et al., 2015; Dos Santos et al., 2016; Mirza et al., 2019). Six studies also evaluated PDT as a monotherapy (Lulic et al., 2009; Bassir et al., 2012; Cappuyns et al., 2012; Kolbe et al., 2014; Alvarenga et al., 2019). Although some trials show no significant changes between the test (PDT) and the control (SRP) group outcomes, several studies reported that PDT offered advantages over SRP in the improvement of outcomes (Bassir et al., 2012; Giannelli et al., 2012), especially in the reduction of microbiological parameters (Mongardini et al., 2014). In accompaniment with this statement, 48 articles (88.88%) identified PDT as complementary to the conventional SRP method (Braun et al., 2008; Campos et al., 2013; Joseph et al., 2016; Raut et al., 2018). However, the importance of PDT in this context is still open to question (Hill et al., 2019). None of the studies reported any side effects for PDT (Giannelli et al., 2012; Alwaeli et al., 2013; Hill et al., 2019).

### 5.5 Effect of PDT on periodontal and clinical parameters

In this review, PPD, CAL, PI, GI, and bacterial count in the involvement sites were defined as the primary output of periodontal parameters that were investigated in almost all studies. BOP, gingival bleeding index (GBI), relative attachment level (RAL), sulcus bleeding index, full-mouth bleeding score (FMBS), and full-mouth plaque score (FMPS), etc. were considered the secondary output of periodontal parameters. Changes in PPD, CAL, and BOP levels, followed by PI, GI, and gingival recession (RC), were the parameters examined most often in almost all trials. These parameters were measured during different periods of time from baseline to 4 years (Giannelli et al., 2015). Levels of CAL as a periodontal marker (Bassir et al., 2012; Aylıkcı and Çolak, 2013; Campos et al., 2013; Betsy et al., 2014; Giannelli et al., 2015; Grzech-Leśniak et al., 2018; Raut et al., 2018; Gandhi et al., 2019) and criterion for the effectiveness of periodontal therapy were reported to show significant improvement (*p*-value ≤0.05). According to Table 3, most studies showed that PPD was significantly decreased following treatment by PDT (*p*-value ≤0.05) (Braun et al., 2008; Bassir et al., 2012; Cappuyns et al., 2012; Alwaeli et al., 2013; Aylıkcı and Çolak, 2013; Campos et al., 2013; Betsy et al., 2014; Grzech-Leśniak et al., 2018; Raut et al., 2018; Vohra et al., 2018; AlAhmari et al., 2019; Gandhi et al., 2019; Grzech-Lesniak et al., 2019; Harmouche et al., 2019; Joshi et al., 2020) and BOP (Braun et al., 2008; Bassir et al., 2012; Cappuyns et al., 2012; Alwaeli et al., 2013; Pulikkotil et al., 2016; Raut et al., 2018). PDT demonstrated no significant effect on HbA1c levels (Barbosa et al., 2018; Mirza et al., 2019), whilst a number of trials showed a reduction of polymorphonuclear leukocytes (PMNs) in the gingival crevicular fluid (GCF) obtained from volunteers following treatment by PDT (Giannelli et al., 2012; Pourabbas et al., 2014; Giannelli et al., 2018; Raut et al., 2018). Significant improvement in the periodontal and clinical parameters was observed in the test group in contrast to the control group (Shingnapurkar et al., 2016).

### 5.6 Effect of PDT on microbiological parameters

**FIGURE 5:**
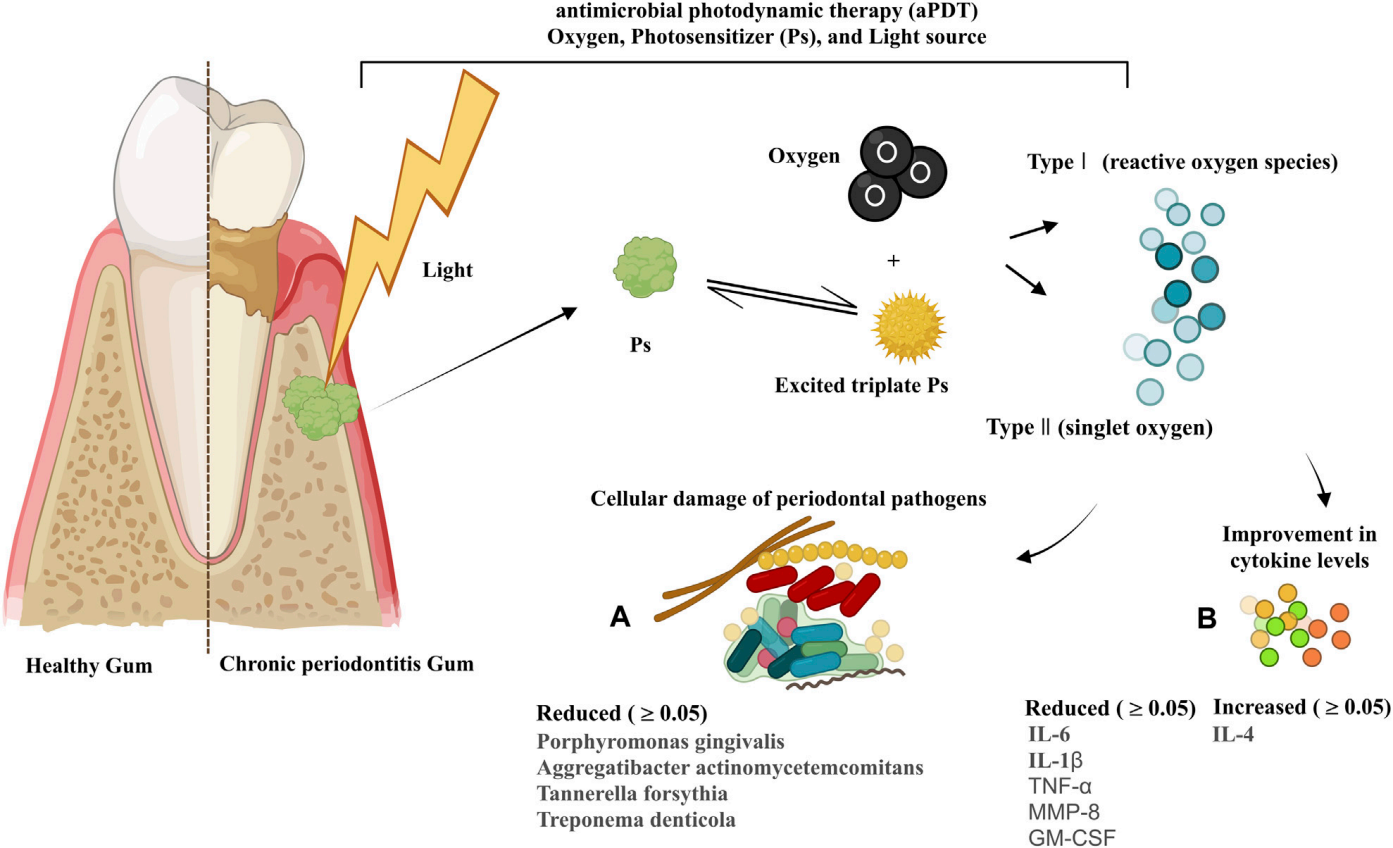
Mode of action of PDT and its components in chronic periodontitis; changes in the profiles of periodontal pathogens **(A)** and cytokines **(B)** following PDT treatment by trials during 2008–2023.

Thirty-one studies performed microbiological analysis, among which 24 trials indicated that PDT is able to significantly reduce periodontal pathogens such as *P. gingivalis* (Pg), *A. actinomycetemcomitans* (A.a), *Tannerella forsythia* (*T. forsythia*; Tf), *Treponema denticola* (*T. denticola*; Td), *Eubacterium nodatum* (*E. nodatum*; En), *Prevotella intermedia* (*P. intermedia*; Pi), *Fusobacterium periodonticum* (*F. periodonticum*), and *Prevotella nigrescens* (*P. nigrescens*; Pn) at 2 months, 3 months, and 6 months after the treatment (Figure 5). Other bacteria examined after PDT treatment included *Peptostreptococcus micros* (*P. micros*), *Campylobacter rectus* (*c. rectus*), *C. gingivalis* (*Capnocytophaga gingivalis*), *E. nodatum* (*E. nodatum*), *Eikenella corrodens* (*E. corrodens*), *Capnocytophaga* spp, *Veillonella parvula* (*V. parvula*), *Parvimonas micra* (*P. micra*), and *F. nucleatum*. In studies by Raut et al. (2018), Campos et al. (2013), and Srikanth et al. (2015), the exact bacterial species evaluated were not specified. The microbial profile showed significant changes in the red complex microbial population in line with Giannelli et al. that showed the load of spirochetes, bacilli, and cocci in patients receiving PDT + SRP (test group) was significantly reduced compared to those receiving only SRP (control group) at the 1- and 4-year follow-ups (Giannelli et al., 2012). However, several studies state there was no significant difference between the treatment and control groups in bacterial levels during longer follow-up periods (*p*-value ≥0.05) (Christodoulides et al., 2008; AlAhmari et al., 2019; Karmakar et al., 2021).

Various methods, including real-time PCR using specific primers for each bacterial species [nine studies (Cappuyns et al., 2012; Kolbe et al., 2014; Pulikkotil et al., 2016; Segarra‐Vidal et al., 2017; Grzech-Leśniak et al., 2018; Raut et al., 2018; AlAhmari et al., 2019; Grzech-Lesniak et al., 2019; Hill et al., 2019)], cytofluorescent staining using the LIVE/DEAD Bac-the Light™ bacterial viability kit [three studies (Giannelli et al., 2012; Giannelli et al., 2015; Srikanth et al., 2015)], bacterial culture and the plate count method [three studies (Raut et al., 2018; Gandhi et al., 2019; Pooja and Mannava, 2020b)], DNA-DNA hybridization assay [one study (de Melo Soares et al., 2019)], and microDent system [one study (Polansky et al., 2009)] were used to detect periodontal pathogens. Samples in the trials were collected from the deepest area of the periodontal pocket (sub-gingiva) (Cappuyns et al., 2012; Pulikkotil et al., 2016; AlAhmari et al., 2019; de Melo Soares et al., 2019) and supra-gingival part of the teeth (Srikanth et al., 2015) by placing sterile paper points in the bottom of pockets for 10 s–30 s and transferring them into the sterile containers that contained PBS (Raut et al., 2018), reduced transport fluid, buffer solution (de Melo Soares et al., 2019), or Robertsons cooked meat with solidified 1% agar (Srikanth et al., 2015; Gandhi et al., 2019). The samples were stored at −20°C to −80°C until processing for genomic analysis or microbial culture in the laboratory.

### 5.7 Effect of PDT on immunological parameters

Eleven studies evaluated immunological parameters, following the collection of GCF using sterile paper points that were placed in the subgingival area of the plaques for 10–30 s. Most of these studies used enzyme-linked immunosorbent assay (ELISA) (Queiroz et al., 2013; Pourabbas et al., 2014; Goh et al., 2017; Segarra‐Vidal et al., 2017; de Melo Soares et al., 2019; Niazi et al., 2020), and only one study used high-sensitivity human cytokine 10-plexi (Kolbe et al., 2014) to measure the levels of cytokines in GCF. All studies mentioned that a calibrated electronic tool was used to measure the volume of GCF. Six studies reported a significant effect of PDT on the reduction of interleukin-6 (IL-6), interleukin-1 beta (IL-1β), tumor necrosis factor α (TNFα), matrix metalloproteinase-8 (MMP-8) (*p*-value ≤0.05), and granulocyte–macrophage colony-stimulating factor (GM-CSF) levels and a significant effect on the increase in the interleukin-4 (IL-4) level (*p*-value ≤0.05) (Figure 5) (Queiroz et al., 2013; Kolbe et al., 2014; Niazi et al., 2020). Other cytokines and immunological parameters measured by the trials were interleukin-8 (IL-8), interleukin-10 (IL-10), interleukin-12 (IL-12), interleukin-13 (IL-13), interferon-γ (IFN-γ), MMP-9, receptor activator of nuclear factor-kappa-Β ligand (RANK-L), and osteoprotegerin decoy receptor (OPG).

### 5.8 Effect of PDT on other parameters (halitosis, teeth injury, and LDH level)

According to the trials, PDT did not show any effects on teeth injury or lactate dehydrogenase (LDH) levels (Srikanth et al., 2015; Grzech-Leśniak et al., 2018). However, the association between periodontal disease and bad breath or halitosis related to poor oral hygiene is indeed debated (Aylıkcı and Çolak, 2013). Betsy et al. (2014) showed that PDT had transient effectiveness on halitosis.

## 6 Discussion

In the current study, 53 clinical trials during 2008–2023 were reviewed. Risk factors such as smoking, diabetes, and obesity not only increase the chances of developing periodontal disease but can also influence the outcome of the treatment process. Therefore, studies with such a situation were excluded from the present systematic review in order to examine the exact effect of photodynamic therapy on the treatment process by considering the conditions of normal patients with periodontitis.

The overall results of the present systematic review showed that PDT treatment could improve several periodontal parameters, including BOP, CAL, PI, GI, and PPD, change the profile of inflammatory and anti-inflammatory cytokines in favor of patient recovery, and significantly reduce the red complex (usually *P. gingivalis*, *T. denticola*, and *T. forsythia*) and blue complex of periodonto-pathogens (*Actinomyces* species) (Mongardini et al., 2014). Evaluation of periodontal parameters, blood cell count (PMN and RBC, especially leukocytes), and bacterial and cytokine profiles will give helpful guidance about the status of periodontal disease (Giannelli et al., 2012). Therefore, any change in these parameters can relate to changes in the severity of periodontal inflammation and bleeding in the oral cavity. Moreover, because leukocytes are considered sources of toxic products such as reactive oxygen species (ROS), inflammatory mediators, and matrix-degrading enzymes, which aggravate inflammation, it seems that reduction of leukocyte levels can help to repair the damaged periodontal tissues (Giannelli et al., 2012; Giannelli et al., 2015).

PDT is a chemical process that produces exogenous ROS in the presence of oxygen when PS is placed in the target area. ROS are the main factor in the effectiveness of the PDT mode of action by destroying abnormal cancer cells (Zheng et al., 2023) and pathogenic bacteria (Ghorbani et al., 2018) that are specifically targeted to be destroyed. On the other hand, under normal conditions, 90% of the ROS in the body are produced by the mitochondrial electron chain in response to hypoxia, ischemia, aging, etc. The presence of endogenous ROS is harmful to the body; thus, 90% of them are reduced to water by cytochrome oxidase. Endogenous ROS are under the control and regulation of mitochondrial membrane potential (MMP) (Sarniak et al., 2016). The question of whether the ROS formed by PDT aggravate inflammation may arise. PDT has the ability to intensify endogenous ROS by affecting MMP, but in general, this ability of exogenous ROS is not impressive, and few studies have addressed this issue (Zhang et al., 2020). More studies are needed. Moreover, the very low half-life of ROS, which is approximately 0.01–0.04 μs in a diffusion distance range of 0.01–0.02 μm (Gunaydin et al., 2021), is very short to trigger an inflammatory response. Therefore, ROS disappear quickly. For this reason, photodynamic therapy is very specific.

Mineralized residual deposits and dental plaques on the root surfaces are believed to be the main cause of bacterial attachment in periodontal lesions by acting as a reservoir for the recurrence of the disease and progression of periodontitis and tooth loss (Bassir et al., 2012; Campos et al., 2013; Raut et al., 2018; Vohra et al., 2018; de Melo Soares et al., 2019). Restoring the compromised periodontal tissues and providing suitable and durable conditions are the primary aims of periodontal therapy (Kotsilkov and Popova, 2010; Russo et al., 2016). Conventional methods such as SRP and antibiotic therapy are currently used for periodontal therapy. Although SRP is still the gold standard for periodontal therapy and pocket reduction therapy, Choi et al. believed that SRP may help bacteria penetrate deeper into the sub-epithelial spaces (Choi et al., 2014). Furthermore, the frequent need for using SRP, the amount of treatment time required (Russo et al., 2016), and the lack of accessibility of SRP into the deep periodontal pockets (≥5 mm) to remove bacterial deposits and toxins (Malgikar et al., 2016) have led to a search for novel strategies to control biofilm formation (Grzech-Leśniak et al., 2018; Gandhi et al., 2019). The success of a periodontal treatment depends on the complete removal of the subgingival biofilm and the eradication of bacteria from the root surfaces (Grzech-Lesniak et al., 2019). Limitations associated with the use of antibiotics in periodontal therapy are reported as allergic reactions, poor accessibility of antibiotics to sub-gingival plaque, antibiotic-resistant bacterial emergence, discoloration of teeth, and gastrointestinal disorders (Socransky and Haffajee, 2000; Bassir et al., 2012; Raut et al., 2018). Studies have shown that the administration of only doxycycline or minocycline did not show any improvements in PPD reduction and CAL gain (Tomasi et al., 2008; Kolbe et al., 2014).

The main side effects of PDT in the oral cavity were reported as burning, pain, and edema (Sharma et al., 2022). The results of the present review showed no emergence of strains resistant to toxic reactive oxygen mediators of PDT (Raut et al., 2018), no cytotoxicity for host cells (Srikanth et al., 2015), and patients undergoing PDT (with or without anesthesia) rarely experience discomfort, which reflects the safety and tolerability of PDT (Giannelli et al., 2012).

In addition to the antimicrobial effects of PDT, a concern about their effect on periodontal tissue and oral microbiota must be clarified. High-level lasers cause thermal damage to periodontal tissue and lead to excessive ablation, root, pulp, and tooth necrosis (Takasaki et al., 2009). Studies have shown that PDT as a low-level laser not only selectively destroys pathogens without affecting the periodontal tissue (Andersen et al., 2007; Christodoulides et al., 2008; Qin et al., 2008; Takasaki et al., 2009) but also improves the blood flow of the gum tissue and increases oxygen delivery to the tissue in inflammatory conditions (Raghavendra et al., 2009). The diode wavelength of recent lasers and their short radiation time do not interact with the periodontal tissue, except in the case of longer radiation duration, which may cause thermal damage to the pulp and teeth due to radiation. Due to the toxicity potential of PS for periodontal tissue, it is recommended to aspirate all the dye from the periodontal pocket after a PDT procedure (Takasaki et al., 2009). According to Hamblin et al., PDF does not have any side effects on the normal and beneficial microflora in other parts of the mouth (Hamblin and Hasan, 2004; Jori et al., 2006). This adds to the superiority and credibility of PDT over antimicrobial agents such as antibiotics because antibiotics destroy all beneficial and pathogenic bacteria without distinguishing between the microbial population in the oral cavity, but PDT targets only the target area due to local radiation and the short half-life of ROS (Gilaberte et al., 2021).

According to the Theodor et al. study, a combination of PDT with antibiotics (metronidazole + amoxicillin) in adjunct to SRP can reduce PPD markers more effectively than SRP alone or antibiotics alone (Pooja and Mannava, 2020b). Therefore, PDT cannot be replaced by antibiotic therapy, but caution is required regarding the development of resistant strains during this treatment method (Bundidpun et al., 2018).

PDT is able to reduce BOP levels, leading to a reduction in inflammation and healing of local periodontal wounds (Campos et al., 2013; Barbosa et al., 2016). Therefore, the absence or reduction of BOP can be directly associated with periodontal stability, and the presence of BOP can be related to the disease progression. PPD and CAL are indicators of periodontal tissue damage and the effectiveness of treatment (Vohra et al., 2018). Because PPD changes can occur in response to any factor, analyzing the clinical attachment level by measuring the distance from the point under examination to the bottom of the periodontal pocket is the gold standard of an appropriate indicator. As dental plaque is the major contributing factor in periodontitis, control of supra-gingival biofilm and PI values is important in improving PPD (Bassir et al., 2012; Gandhi et al., 2019).

Although the anti-cancer effects of PDT have been studied, a few studies evaluated the effect of PDT on the immune system in oral infection. PDT leads to the enhancement of apoptosis, improvement of the function of damaged tissue, and establishment of homeostasis between inflammatory and anti-inflammatory cytokines (Falk-Mahapatra and Gollnick, 2020; Mosaddad et al., 2023). Periodontal pathogens can escape from the host immune system and inactivation mediated-antibiotics by penetrating into the epithelial cells and dental pockets, which helps them to re-grow and cause chronic disease (Giannelli et al., 2015). *Salvadora persica* is an herbal gel with silica, sodium bicarbonate, and tannic acid compounds used to decrease dental plaque on root surfaces, bleeding, and inflammatory mediators, as well as to maintain a normal pH in the mouth. This herbal medication can also reduce periodontal pathogens such as *A. actinomycetemcomitans*, *P. gingivalis*, and *S. mutans* (Niazi et al., 2020). LPS released from the Gram-negative bacteria enables bacterial attachment to root surfaces and their survival even after SRP, leading to chronic inflammation (Giannelli et al., 2012). In this context, TBO has stronger bactericidal properties than MB due to the stronger binding of TBO to the bacterial LPS. This also makes Gram-negative bacteria more sensitive to inactivation by PDT (Raut et al., 2018). Moreover, the significant decrease in PMNs of the oral cavity, usually seen after PDT, could be related to the radiation-induced inactivation of LPS.

Chronic periodontitis patients show an increase in the TNF-α, IL-6, IL-1B, and RANK-L levels and a decrease in OPG. RANK-L and OPG are two important factors in the regulation of osteoclastogenesis that can reflect the patient’s condition. PDT is proved to have the potential to suppress inflammation (Huang et al., 2019; Phutim-Mangkhalthon et al., 2020; Deng et al., 2023), pro-inflammatory cytokines and RANK-L (Segarra‐Vidal et al., 2017). IL-6 and TNF-α are pro-inflammatory cytokines that stimulate bone decomposition. PDT decreases these pro-inflammatory cytokines by reducing T-lymphocyte stimulation and interfering with APC stimulatory function (Kolbe et al., 2014; Niazi et al., 2020). IL-1 and MMP-8 are two main immunological factors involved in the tissue destruction of periodontitis. High levels of MMP-8, following SRP, indicate further loss of periodontal tissue, while PDT proved to have the potential to improve it (Queiroz et al., 2013).

The potential reasons for discrepancies or contradictory results among different trials that evaluated the impact of PDT on the treatment of periodontitis may be related to the lack of a standard protocol for PDT, variations in the type and concentration of the photosensitizer, differences in the penetration mode of the photosensitizer, duration of its placement at the site of infection (Segarra‐Vidal et al., 2017), laser-related characteristics (laser device type, output power, wavelength, and irradiation time), type of the studied teeth, frequency and combination using of PDT, and the presence of any medical condition among patients, age, gender, hormonal changes, etc. (Alwaeli et al., 2013; Campos et al., 2013; Bundidpun et al., 2018; Hill et al., 2019). These are potential reasons for any discrepancies or contradictory results observed across different trials.

It is important to use a proper type of photosensitizer to reach desirable outcomes in PDT. Differences in the cell walls of Gram-positive and Gram-negative bacteria (the thickness of peptidoglycan, presence/absence of lipoteichoic acid (LTA), outer membrane, and LPS) influence the permeability rate of photosensitizers and application of PDT in removing these bacteria (Grzech-Leśniak et al., 2018). In this regard, Gram-positive bacteria are eliminated by both types of positive- and negative-charged photosensitizers, while only positive-charged photosensitizers have antibacterial effects on Gram-negative bacteria.

PDT has great anti-biofilm effects on single-rooted teeth; thus, the type of the studied teeth in different trials can also affect the efficacy of the PDT treatment (Alwaeli et al., 2013). Periodontal pockets in multiple-rooted teeth show less response to SRP + PDT treatment than non-molar teeth (Bassir et al., 2012). This is consistent with the Campos et al. study that showed a positive effect of PDT on single-rooted teeth of patients with chronic periodontitis (Campos et al., 2013). Thus, it is recommended that studies performed on multiple-root teeth not be compared with those performed on single-rooted teeth (Dos Santos et al., 2016). No significant difference in microbiological analysis was attributed to the translocation of pathogenic bacteria (Karmakar et al., 2021) and insufficient removal of the biofilm in the root surface by the PDT protocol (Birang et al., 2015).

Single or multiple applications of PDT are another important issue that can result in discrepancies in outcomes. However, some evidence showed the frequency of PDT administration may result in no significant difference between the intervention and control groups. It is suggested that if PDT is repeated in the first week of the intervention, the effect of PDT will be strengthened (Christodoulides et al., 2008). Other RCTs reported that repeated use of PDT, which is also known as the dose effect (Gandhi et al., 2019), along with the SRP can show better outcomes (Barbosa et al., 2016; AlAhmari et al., 2019; Hill et al., 2019; Mirza et al., 2019; Joshi et al., 2020). This is proved by Alwaeli et al. (2013), Campos et al. (2013), Grzech-Leśniak et al. (2018), and Pooja and Mannava (2020b). Furthermore, halitosis, which is characterized by the release of volatile sulfur compounds and bad breath (De Geest et al., 2016), will be significantly improved after several episodes of PDT (Betsy et al., 2014). In contrast to single PDT or single SRP, a combination therapy of PDT with SRP makes significant improvements in the treatment outcome. This claim is supported by studies that show that the use of PDT + SRP causes a larger decrease in the number of plaques ≥5 mm, BOP, and risk of progression to destructive periodontitis and tooth loss than monotherapy with SRP alone (Alwaeli et al., 2013; Campos et al., 2013; Kolbe et al., 2014). Another factor affecting the treatment outcomes is menstruation, which is associated with increased expression of inflammatory cytokines and inflammation in periodontal tissues. Hormonal changes may affect the outcome of SRP + PDT periodontal therapy in trials carried out on women.

## 7 Limitations and further research

There is wide heterogeneity among RCTs due to a lack of standard clinical protocol for photodynamic therapy and small sample sizes, which limits the comparison of RCTs and makes claims about the reliability of PDT for the treatment of oral diseases difficult to quantify. We could have considered the effect of PDT on patients with underlying diseases or special conditions like diabetes, obesity, smokers, etc., but due to the large amount of content that may be beyond the reader’s interest, we excluded them from the present study and will examine those studies in a future review. In addition to superficial localized infection and cancer, the present study showed that PDT is effective on oral diseases like periodontitis, but more trial studies on a larger scale, development of more standardized protocols, novel and various photosensitizers, and efficient delivery strategies are needed.

## 8 Conclusion

The evidence from RCTs has shown that photodynamic therapy was as effective as conventional therapy like SRP in ameliorating clinical symptoms such as PPD and CAL and reducing pathogens and periodontal pockets, especially when used with conventional therapy. Although photodynamic therapy is able to overcome the limitations of the SRP, more large-scale clinical trials are needed. Photodynamic therapy is a promising, adjunctive, and low-cost therapeutic method that is effective in tissue repair, reducing chronic periodontitis, reducing inflammation, and well-tolerated by patients. For these reasons, photodynamic therapy can be used as a potential complementary method to conventional therapy. Moreover, photodynamic therapy, along with SRP, appears to mediate the conditions for periodontal recovery in the long term.
